# Molecular characterization of colorectal mucinous adenocarcinoma and adenocarcinoma, not otherwise specified, identified by multiomic data analysis

**DOI:** 10.3389/fmolb.2023.1150362

**Published:** 2023-04-05

**Authors:** Kailun Xu, Shu Zheng, Baosheng Li, Yingkuan Shao, Xiaoyang Yin

**Affiliations:** ^1^ Department of Breast Surgery and Oncology (Key Laboratory of Cancer Prevention and Intervention, China National Ministry of Education, Key Laboratory of Molecular Biology in Medical Sciences, Zhejiang Province, China), Cancer Institute, The Second Affiliated Hospital, Zhejiang University School of Medicine, Zhejiang Provincial Clinical Research Center for Cancer, Cancer Center of Zhejiang University, Hangzhou, Zhejiang, China; ^2^ Department of Radiation Oncology, Shandong Cancer Hospital and Institute, Shandong First Medical University and Shandong Academy of Medical Sciences, Jinan, Shandong, China

**Keywords:** colorectal cancer, mucinous adenocarcinoma, adenocarcinoma, not otherwise specified, tumor microenvironment, stromal cell, epithelial cell

## Abstract

Adenocarcinoma not otherwise specified (AC) and mucinous adenocarcinoma (MC) have different biological behaviors and clinical features. We utilized our previous proteomic data and public transcriptome, single-cell transcriptome, and spatial transcriptome databases to profile the molecular atlas of the tumor microenvironments of MC, AC, and normal colon tissues. By exploring the general and specific molecular features of AC and MC, we found that AC was immune-active but exposed to a hypoxic microenvironment. MC cells could protect against DNA damage, and the microenvironment was unfavorable to leukocyte transendothelial migration. We identified several potential molecular and cellular targets of AC and MC for future research. We also highlighted that the major difference between AC and MC was not the variety of cell types and functions but possibly cell interactions. Stromal and epithelial cell interactions play important roles in both MC and AC, but different regulatory pathways were involved.

## Introduction

Colorectal cancer (CRC) is the second most common cancer and causes approximately 196,000 deaths each year in China alone (Cao et al., 2021). From 2000 to 2016, the incidence rate and mortality of colorectal cancer have shown an upward trend there. Most colorectal cancers are classified as adenocarcinoma not otherwise specified (AC). Mucinous adenocarcinoma (MC) is characterized by the formation of a tumor composed of >50% mucin and accounts for approximately 10%–15% of all primary CRCs (15% of colon cancer versus 9% of rectal cancer) (Hugen et al., 2015a). AC is arranged in solid masses or small strips with gland cavities or into tubular or gland-like structures. MC shows expansile growth with clusters, strips, or singly arranged tumor cells floating in mucin pools.

Apart from morphological differences, there are different biological behaviors and clinical characteristics between AC and MC (summarized in Supplementary Table S1). Patients with MC are more often correlated with advanced-stage tumors, lymph node metastases, a larger diameter, and localization in the proximal colon (Nozoe et al., 2000; Chiang et al., 2010; Melis et al., 2010). Compared with AC, MC is related to an earlier onset age, a more advanced clinical stage, a higher degree of difficulty in surgery, and higher rates of recurrence and metastasis (Ahnen et al., 2014; Luo et al., 2019). It is difficult to effectively prevent tumor recurrence and progression through various treatments (S. H. Kim et al., 2013). MC patients mostly die of malignant intestinal obstruction (Hugen et al., 2016). Most studies have shown that MC patients often have a lower progression-free survival rate and a shorter median overall survival time (Catalano et al., 2012; Numata et al., 2012; Ott et al., 2018). Unfortunately, MC is not sensitive to chemotherapy and radiotherapy (S. H. Kim et al., 2013; McCawley et al., 2016), and other available treatments—such as targeted molecular therapy, hyperthermic intraperitoneal chemotherapy, and immunotherapy—are also not satisfactory (Luo et al., 2019; Huang et al., 2021).

Regardless of advances in the molecular characterization of CRC, there are still few molecular targets or biomarkers that could be clinically applied to improve individual treatment. Clinical features, including histologic subtype, tumor grade, lymphatic and perineural invasion, tumor budding, and host immune responses, remain the main factors in CRC treatment. Molecular biomarkers (Biller and Schrag, 2021; Ciardiello et al., 2022), such as microsatellite instability (MSI), *BRAF*, *KRAS*, *NRAS*, *RAS*, *HER2*, and *NTRK*, have contributed greatly to CRC treatment. However, they are unable to clarify some malignant events in CRC or explain the different biological behaviors between AC and MC.

Thus, it is important to decipher the molecular signature of AC and MC to improve the individualized management of patients with CRC. It has been documented that MC is related to a higher frequency of *KRAS* and *BRAF* mutations, microsatellite instability, the CpG island methylator phenotype pathway, and a lower frequency of TP53 mutations (Luo et al., 2019), on which the current treatment is mainly based. The MUC (mucin protein) family is aberrantly expressed in colorectal MC, which might be related to tumor invasion, metastasis, and chemoradiotherapy resistance (Reynolds et al., 2019). Targeted drugs for mucins are expected to be sensitive to chemoradiotherapy or immunotherapy and to improve the prognosis of patients with MC. However, there has still been no promising research outcome. Identifying comprehensive molecular characteristics will hopefully provide new strategies.

In this study, we tried to profile the molecular atlas of AC and MC with our previous proteomic data and public transcriptome, single-cell transcriptome, and spatial transcriptome databases. We found that AC was immune-active and anaerobic. MC cells could protect against DNA damage, and the microenvironment was unfavorable to leukocyte transendothelial migration. Stromal and epithelial cell interactions play important roles in both AC and MC, and different regulatory pathways are involved. There were differences in the characteristics of the tumor environments of AC and MC. The major difference was not the variety of cell types and functions but may be cell interactions.

## Materials and methods

### Bioinformatic analysis of the proteomics dataset

The proteomic data were obtained from our previous study (Shao et al., 2022; Siegel et al., 2022). These data were from 16 normal colon tissues, 15 colorectal AC samples, and 15 MC colorectal cancer samples (Supplementary Table S2). Gene set enrichment analysis (GSEA) was performed using the OmicStudio tool (https://www.omicstudio.cn/tool) with hallmark gene sets from the Molecular Signatures Database (MSigDB, c2.cp.kegg.v7.4). Significantly enriched hallmarks were chosen according to the cutoff *p*-value of <0.05. Protein–protein interaction networks functional enrichment analysis by STRING (https://string-db.org/, v11.5) was employed to perform Gene Ontology (GO) analysis, including biological processes (BPs), cellular components (CCs), and molecular functions (MFs), and distribution of tissue expression. Network analysis was performed using Cytoscape v3.7.2 with network data downloaded from STRING. R v3.6.0 was used for statistical analysis.

### Bioinformatic analysis of the single-cell RNA sequencing (scRNA-seq) dataset

Human colorectal cancer scRNA-seq data from a study by Hacohen et al. (Pelka et al., 2021) (GSE178341) were downloaded from GEO. Seurat 3.0 was used to perform data normalization, variable feature finding, data scale, and reduction; after cells were clustered, we used the metadata from the paper to annotate the single cell type. NicheNet analysis (Browaeys et al., 2020) was performed using tools at saeyslab/nichenetr: NicheNet: predict active ligand–target links between interacting cells (github.com).

### Bioinformatic analysis of the spatial transcriptome dataset

Human colorectal cancer spatial transcriptome data from Hu et al. (Peng et al., 2022) were downloaded. Dimensionality reduction and clustering and integration of the spatial transcriptome data with the scRNA-seq data from GSE178341 were used to perform cell annotation.

## Results

### Common proteomic characteristics between two types of colorectal adenocarcinomas and normal colon

**FIGURE 1:**
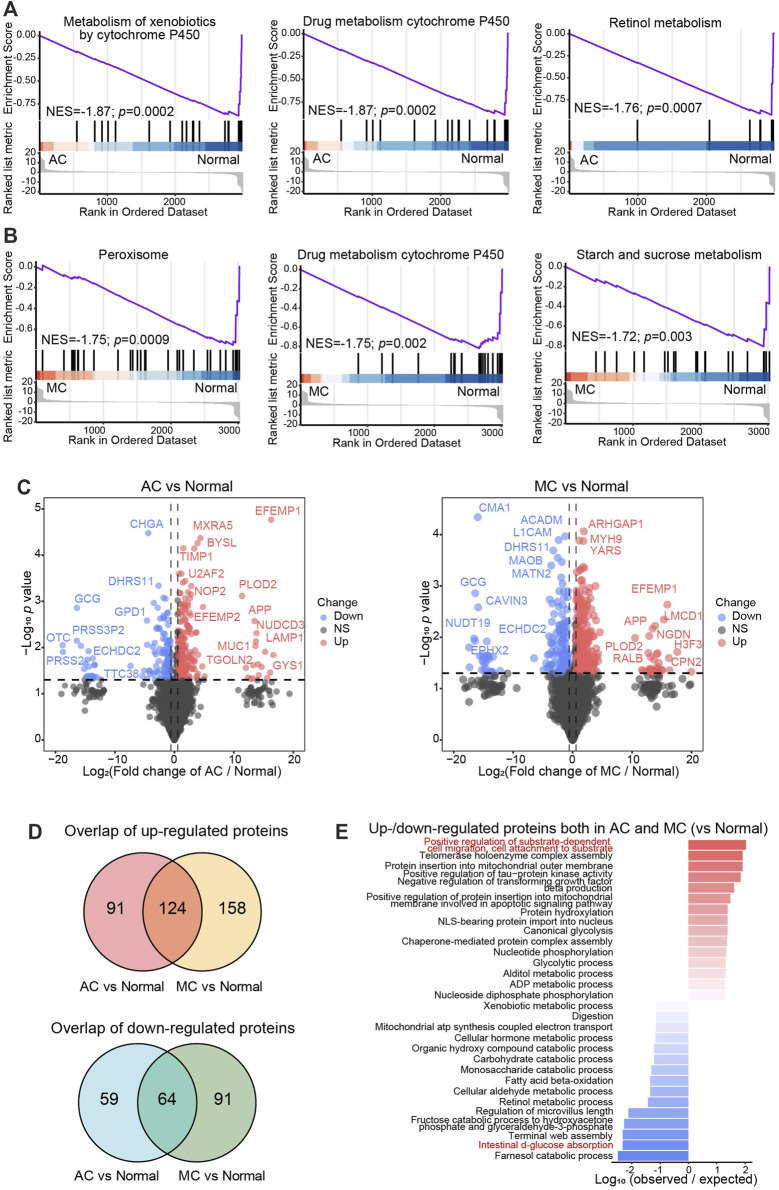
Common proteomics characteristics between two types of colorectal adenocarcinomas and normal colon. A–B: Gene set enrichment analysis (GSEA) showed the top three significantly enriched hallmarks between AC and normal tissue **(A)**, and MC and normal tissue **(B)**. NES: normal enrichment score. **(C)** Volcano plots showed the differentially expressed proteins between AC and normal tissue (left), and MC and normal tissue (right). Cutoff: fold change >1.5, *p* < 0.05. **(D)** Venn diagrams exhibited the overlap up- (upper) or down- (lower) regulated differentially expressed protein numbers between AC and normal tissue, and MC and normal tissue. **(E)** Bar plot showed the top -15 biological processes enriched with the overlap up- or downregulated differentially expressed proteins according to log_10_ (observed/expected proteins).

We analyzed proteomics data from our previous study to profile the protein characteristics of AC, MC, and normal colon samples. First, GSEA was performed for the AC vs. normal colon groups and MC vs. normal colon groups to determine whether any signatures were enriched in the specific groups. The results indicated that the signatures were similar when the MC and AC groups were compared to the normal colon group. The top three signatures enriched in the AC group (vs. the normal group) were related to peroxisomes, drug metabolism involving cytochrome P450, and starch and sucrose metabolism (Figure 1A). The top three signatures enriched in the MC group (vs. the normal group) were associated with the metabolism of xenobiotics by cytochrome P450, drug metabolism involving cytochrome P450, and retinol metabolism (Figure 1B), which were all downregulated.

To further investigate the molecular characteristics of different pathologies with that of normal tissue, we compared the differentially expressed proteins between the AC group and the normal colon group and between the MC group and the normal colon group (fold change >1.5, *p*-value <0.05, Figure 1C, Supplementary Table S3). There were 215 proteins that were upregulated and 123 proteins that were downregulated in the AC group compared to the normal colon group, and 282 proteins that were upregulated and 155 proteins that were downregulated in the MC group compared to the normal colon group (Figure 1D). A total of 124 upregulated proteins and 64 downregulated proteins overlapped. The overlapping upregulated proteins were mostly related to the positive regulation of substrate-dependent cell migration and cell attachment to substrate (Figure 1E). The biological process of intestinal d-glucose absorption was enriched in the overlapping downregulated proteins. These findings indicate dysfunction of gut cells in CRC, which results in cachexia.

Collectively, we found that drug metabolism involving cytochrome P450 was inactive, and substrate-dependent cell migration and cell attachment to substrate processes were important for both the AC and MC groups.

### Proteomic differences between two types of colorectal adenocarcinomas and normal colon tissue

**FIGURE 2:**
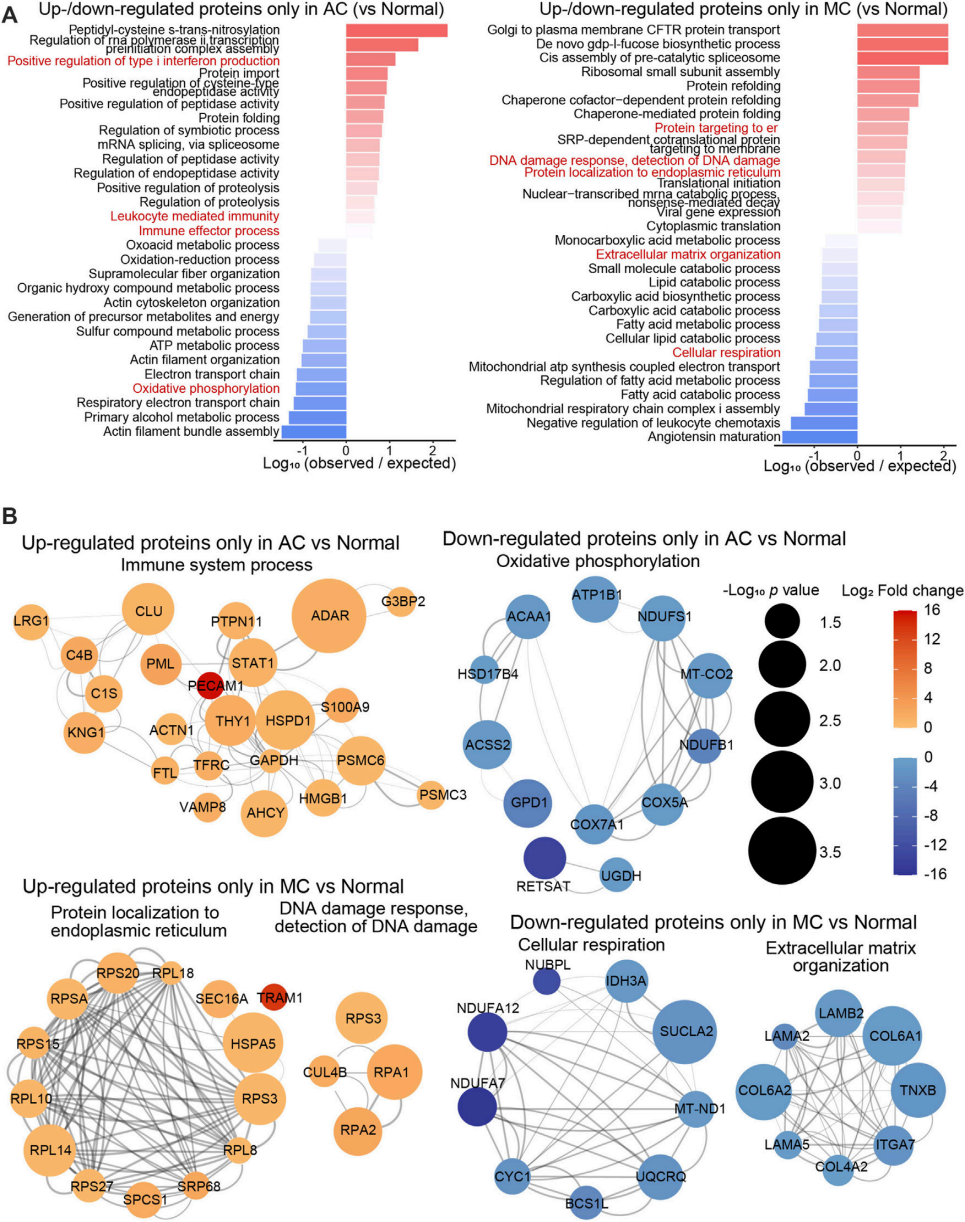
Proteomics difference between two types of colorectal adenocarcinomas and normal colon. **(A)** Bar plots showed the top 15 biological processes enriched with up- or downregulated differentially expressed proteins only in AC (left) or in MC (right). **(B)** Networks presented proteins of partial representative biological processes. Color bar: -log_10_ (protein expression *p*-value of AC/normal or MC/normal). Circle size: -log_10_ (protein expression fold change of AC/normal or MC/normal).

To investigate differences in the proteomics between the two types of CRC and normal colon tissue, the biological processes of proteins up- or downregulated only in the AC or MC group compared with the normal colon group were enriched (Figure 2A), and proteins of some representative biological processes were assessed by network analysis (Figure 2B). The biological processes that positively regulate type I interferon production—leukocyte-mediated immunity and immune effector processes—were enriched in the proteins that were upregulated only in the AC group. Oxidative phosphorylation and other respiratory electron transport chain-related biological processes were enriched in the proteins that were downregulated only in the AC group. Among them, PECAM1 (platelet and endothelial cell adhesion molecule 1, also known as CD31) was one of the most significantly upregulated proteins found only in the AC group, and RETSAT (retinol saturase) was one of the most significantly downregulated proteins. Moreover, biological processes, including protein localization to the endoplasmic reticulum, DNA damage response, and detection of DNA damage, were enriched in the proteins upregulated only in the MC group. Extracellular matrix organization- and cellular respiration-related proteins were downregulated in the MC group. TRAM1 (translocation-associated membrane protein 1) and NDUFA7, NDUFA12, and NUBPL were some of the most significantly up/downregulated proteins found only in MC.

**FIGURE 3:**
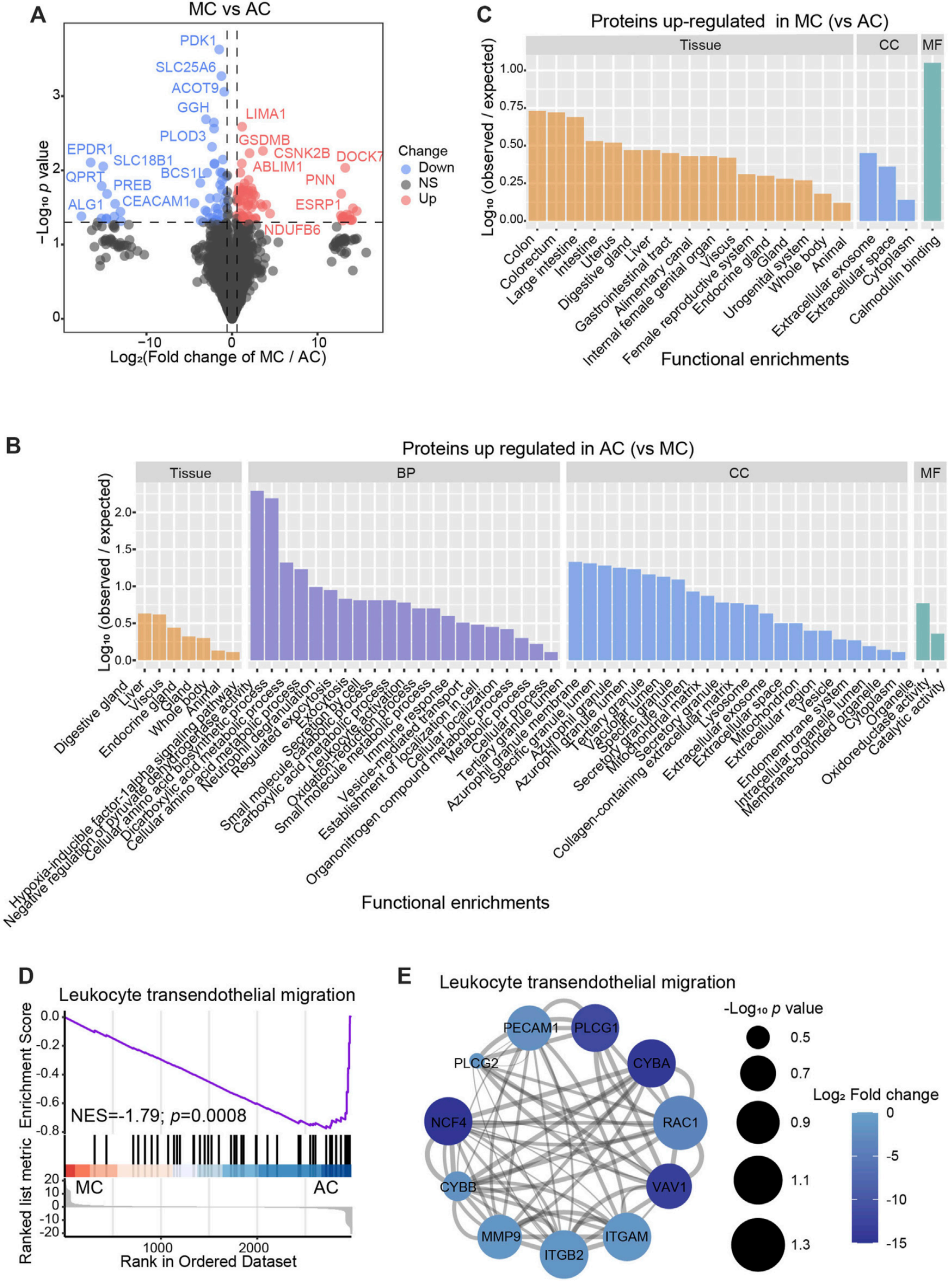
Proteomics difference between two types of colorectal adenocarcinomas. **(A)** Volcano plots showed the differentially expressed proteins between AC and MC. Cutoff: fold change >1.5, *p* < 0.05. B–C: Bar plots showed the distribution of tissue expression and gene ontology (GO) analysis, including biological process (BP), cellular component (CC), and molecular function (MF), which was enriched with proteins upregulated in AC **(B)** or MC **(C)**. **(D)** Gene set enrichment analysis (GSEA) showed the top significantly enriched hallmark between AC and MC. **(E)** Networks presented proteins of the top GSEA between AC and MC. Color bar: -log_10_ (protein expression *p*-value of AC and MC). Circle size: -log_10_ (protein expression fold change of AC/MC).

To gain further insight into the different protein features of AC and MC, we identified the differentially expressed proteins between the AC and MC groups (fold change >1.5, *p*-value <0.05, Figure 3A). The proteins upregulated in the AC and MC groups were mainly located in the digestive gland, whereas proteins upregulated in the MC group were also found to be present in the internal female genital organ. This was another organ system, which includes the ovary, that was at high risk of the occurrence of primary mucinous carcinoma and of colorectal mucinous carcinoma metastasis (Figures 3B, C). We inferred to a certain degree the homogeneity of MC in the digestive tract and internal female genital organs. The hypoxia-inducible factor-1 alpha signaling pathway, various metabolic processes, and leukocyte activation were significantly enriched in the proteins upregulated in the AC group compared to the MC group. For the cellular component, the proteins upregulated in the AC group were mainly located in the tertiary granule lumen. Moreover, proteins upregulated in the MC group were mostly secreted extracellularly with exosomes, which suggests active crosstalk in the tumor microenvironment. The leukocyte transendothelial migration pathway was the most significantly enriched pathway, as determined by GSEA between the AC and MC groups (Figure 3D). Compared with that in the AC group, the leukocyte transendothelial migration pathway was inhibited in the MC group; the involved factors include VAV1 (vav guanine nucleotide exchange factor 1), NCF4 (neutrophil cytosolic factor 4), MMP9 (matrix metalloproteinase-9), ITGB2 (integrin beta-2), ITGAM (integrin alpha-M), and PECAM1 (Figure 3E).

In this section, we compared the differences in proteomics between two types of colorectal adenocarcinomas and normal colon tissues, with a direct comparison between the two types of CRC. AC was immune-active but was exposed to a hypoxic microenvironment. MC cells could protect from DNA damage, and the microenvironment was unfavorable to leukocyte transendothelial migration.

### Single-cell transcriptome atlas of human colorectal adenocarcinoma not otherwise specified, mucinous adenocarcinoma, and normal colon tissue

**FIGURE 4:**
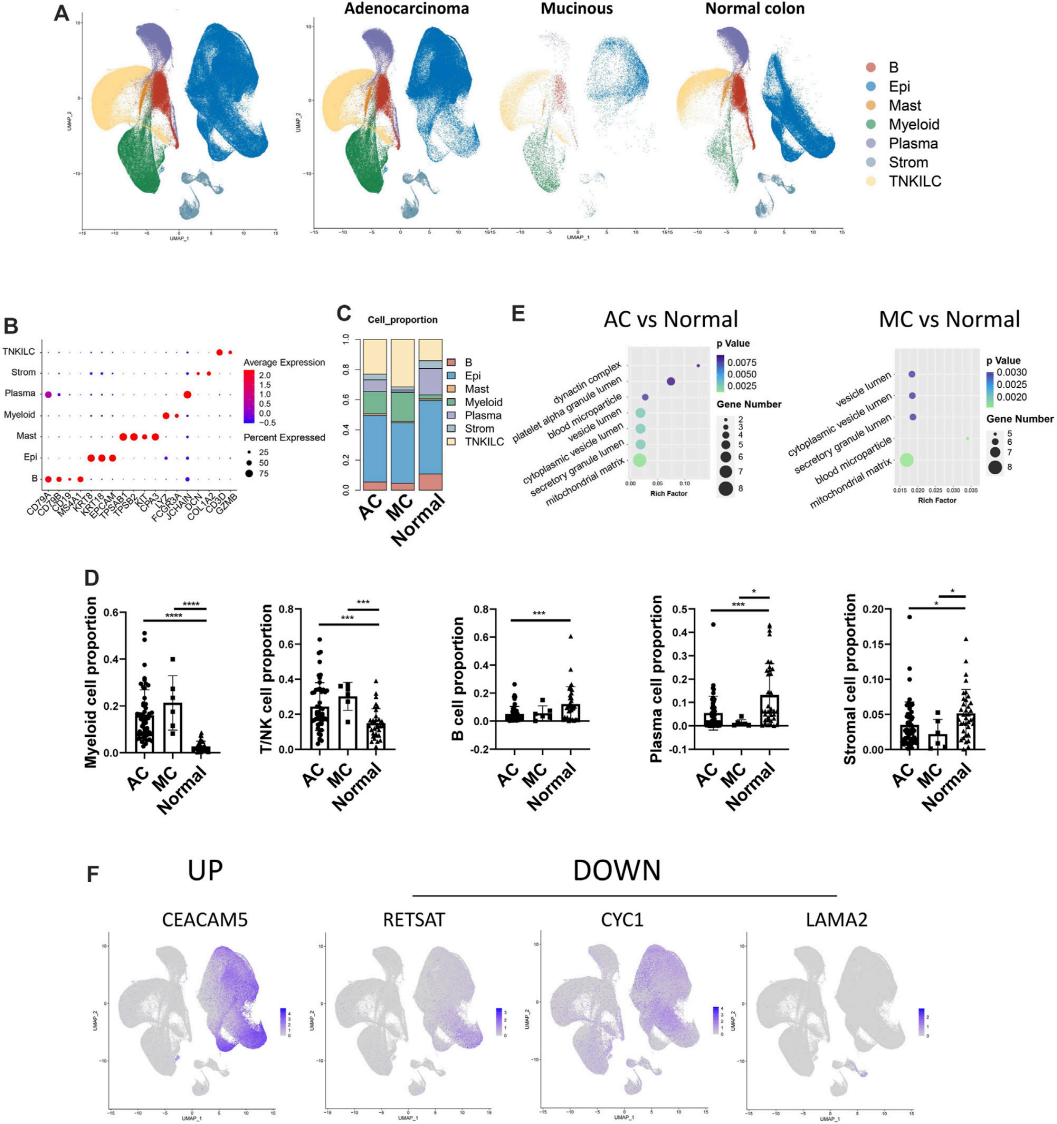
Single-cell transcriptome atlas of human colorectal adenocarcinoma not otherwise specified, mucinous adenocarcinoma, and normal colon. **(A)** Coarse cell annotation of scRNA-seq data of two types of colorectal adenocarcinomas and normal colon. **(B)** Marker genes of coarse cell type of scRNA-seq data on two types of colorectal adenocarcinomas and normal colon. **(C)** Coarse cell type proportion of two types of colorectal adenocarcinomas and normal colon. **(D)** Bar plot showing the significance of different cell-type proportions in two types of colorectal adenocarcinomas and normal colon. **(E)** BP pathways which were enriched in DEGs from AC and normal tissue, and MC and normal tissue. **(F)** Feature plot of mRNA in scRNA-seq data corresponding to differentially expressed proteins found in proteomics data.

We analyzed the GSE178341 scRNA-seq dataset, which includes 36 normal colon tissues, 55 AC, and 7 MC (Supplementary Table S2). We selected min.cell = 3 and min. feature = 200 when creating SeuratObject. Then, we used the metadata provided to perform the cell type annotation. After data normalization, we clustered cells based on significant principal components. There were seven major cell types, including B cells (*n* = 25,455) that expressed CD79A, CD79B, CD19, and MS4A1; epithelial cells (*n* = 163,146), expressing KRT8, KRT18, and EPCAM; mast cells (*n* = 3767), expressing TPSAB1, TPSB2, KIT, and CPA3; myeloid cells (*n* = 40,420), expressing LYZ and FCGR3A; plasma cells (n = 37,513), expressing JCHAIN; stromal cells (*n* = 15,041), expressing DCN and COL1A2; and T/NK cells (*n* = 74,438) that expressed CD3D and GZMB (Figure 4A). The defined marker genes are shown in Figure 4B. We then analyzed different cell proportions in MC, AC, and adjacent normal tissue (Figures 4C, D). Myeloid cells and T/NK cells were significantly increased in both AC and MC tissues. Plasma cells and stromal cells were significantly decreased in both AC and MC tissues. B cells in the AC and MC groups were also decreased but were only significantly decreased in the AC group. These cell proportion differences suggest a remodeling of the tumor microenvironment. However, we did not find a significant difference between the AC and MC groups, which might be due to the coarse cell annotation. Further cell subtype proportion analysis is needed to investigate the differences in cell type proportions between AC and MC.

Differentially expressed genes (DEGs) between MC and adjacent normal tissue and between AC and adjacent normal tissue were identified, and GO enrichment analysis was performed to analyze the involved pathways (Figure 4E). Most of the enriched pathways of the DEGs between MC and adjacent normal tissue and between AC and adjacent normal tissue overlapped. The dynactin complex pathway was only enriched in DEGs between AC and normal tissue. In MC tissue, we did not observe an enrichment of the dynactin pathway, which was consistent with our finding that AC tissue had a more favorable environment for cell migration than MC tissue. The vesicle lumen was the pathway that was most significantly enriched in DEGs between MC and normal colon tissue. This finding is consistent with the results from the proteomic analysis, which indicate that proteins upregulated in MC tissue would mostly be related to extracellular secretion.

We then investigated the expression of the differentially expressed proteins that we found in the proteomics data in the single-cell data to determine whether there was any overlap and in which cluster they were expressed. We selected the most variable differentially expressed proteins (fold change >1.5, *p*-value <0.05) among AC, MC, and normal colon tissues. These overlapping molecules might play essential roles in the special pathology of CRC and be potential targets for treatment. To investigate in which cell type these markers were expressed, we used feature plots to show the expression pattern of these markers (Figure 4F, Supplementary Figure S1A). Among the 30 most variable proteins, 18 corresponding mRNAs were detected in the scRNA-seq data. Of these, four mRNAs (CEACAM5, RETSAT, CYC1, and LAMA2) showed specific expression in one cell type. In our protein data, CEA cell adhesion molecule 5 (CEACAM5) was upregulated, while retinol saturase (RETSAT) was downregulated in the AC group compared with the normal colon group. Cytochrome (CYC1) was downregulated in the MC group compared with the normal colon group. These three corresponding mRNAs were mainly expressed in epithelial cells in the scRNA-seq data. Laminin subunit alpha 2 (LAMA2) was downregulated in the MC group compared with the normal colon group and was mainly expressed in stromal cells. *CEACAM5* is the coding gene of CEA that is expressed in many epithelial malignancies, including colorectal cancer (DeLucia et al., 2021). *CYC1* is a metabolism-related gene that is downregulated in colorectal cancer by several miRNAs after m6A modification, thereby reprogramming the mitochondrial metabolism in colorectal cancer (Yao et al., 2022). These findings suggest that the most variable proteins in AC and MC, compared to normal colon tissue, are mostly expressed in epithelial and stromal cells, indicating that epithelial and stromal cells might play a key role in CRC.

In this section, we analyzed the variation in cell types and DEGs among AC, MC, and normal colon tissues. The differences in cell type and function between AC and MC tissue were not significant, but we found that epithelial and stromal cells might play a key role in CRC. Further investigation is needed to explore the two cell types and their interactions, which we inferred contributed to the different biological behaviors and clinical outcomes of the two CRC subtypes.

### Epithelial and stromal cell infiltration in colorectal adenocarcinoma not otherwise specified, mucinous adenocarcinoma, and normal colon tissue

**FIGURE 5:**
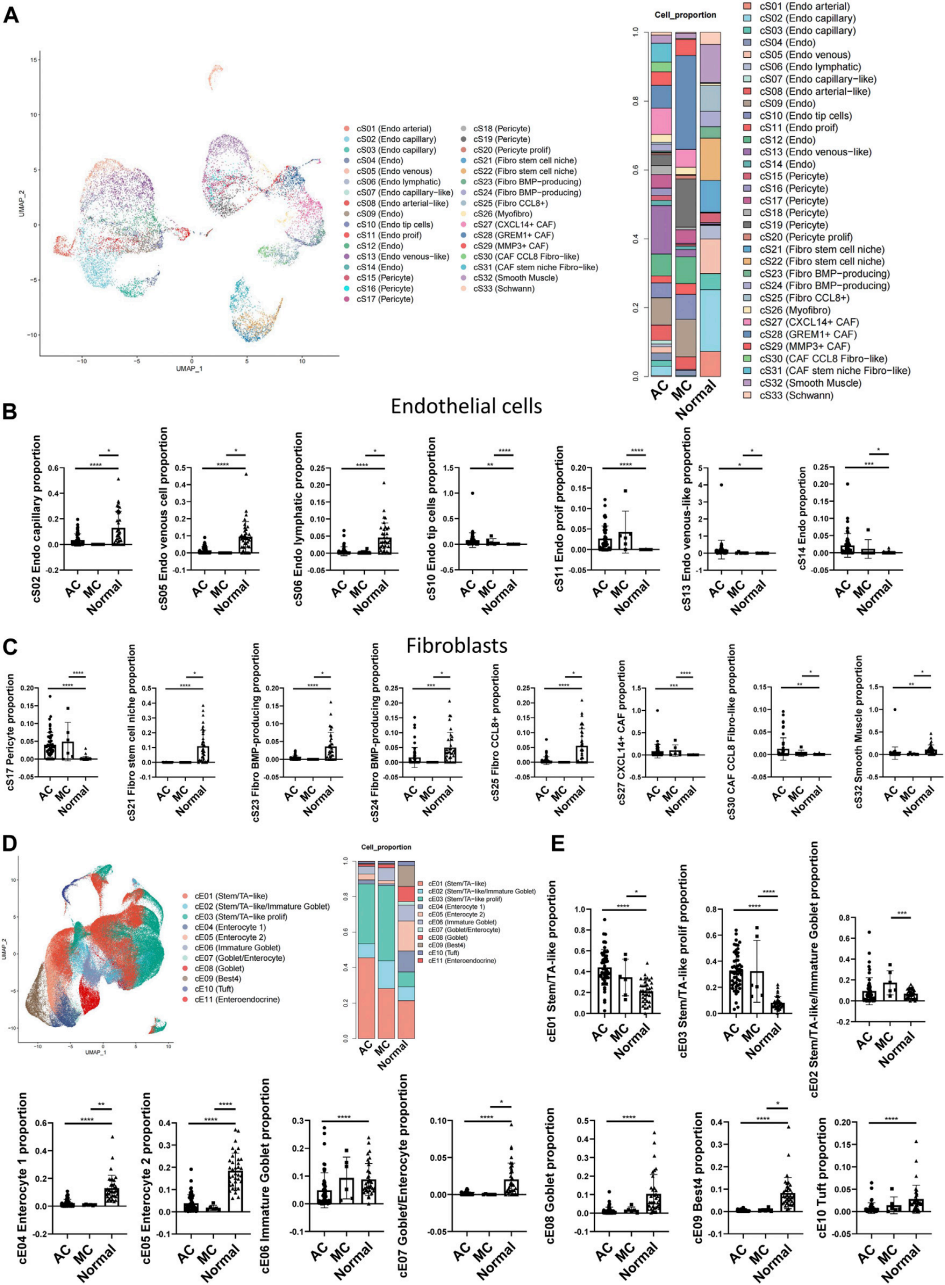
Stromal cell and epithelial cell proportions in human colorectal adenocarcinoma not otherwise specified, mucinous adenocarcinoma, and normal colon. **(A)** Stromal cell subcluster and proportion in human colorectal adenocarcinoma not otherwise specified, mucinous adenocarcinoma, and normal colon. **(B)** Bar plots showing the significance of different endothelial cell proportions in two types of colorectal adenocarcinomas and normal colon. **(C)** Bar plots showing the significance of different fibroblast proportions in two types of colorectal adenocarcinomas and normal colon. **(D)** Epithelial cell subcluster and proportion in human colorectal adenocarcinoma not otherwise specified, mucinous adenocarcinoma, and normal colon. **(E)** Bar plots showing the significance of different epithelial cell proportions in two types of colorectal adenocarcinomas and normal colon.

CRCs, including ACs and MCs, mostly originate from epithelial cells (Joanito et al., 2022). To investigate the potential interaction between epithelial cells and stromal cells, we first analyzed the subcluster of stromal and epithelial cell proportions in AC, MC, and normal tissues (Figures 5A, D). The proliferating endothelial cell proportion was higher in AC and MC tissues than in normal tissue, which indicates angiogenesis in AC and MC tissues (Figure 5B). The proportions of most subtypes of fibroblasts were lower in AC and MC tissues than in normal tissue, whereas the pericyte proportion was higher in AC and MC tissues than in normal tissue. Pericyte–fibroblast transition has been reported to promote tumor growth and metastasis (Hosaka et al., 2016), indicating the importance of angiogenesis in CRC. CXCL14^+^ cancer-associated fibroblasts (CAFs) and CCL8 fibroblast-like cell proportions were also higher in AC and MC tissues than in normal tissue (Figure 5C). The stem-like epithelial cell proportion was higher and the enterocyte and goblet proportions were lower in AC and MC tissues than in normal tissue (Figure 5E). Moreover, the proportions of bestrophin 4-positive (BEST4^+^) cells and Turf cells were lower in AC and MC tissues than in normal tissue. These findings indicate active proliferation and angiogenesis in AC and MC tissues. CXCL14+ CAFs and CCL8 fibroblast-like cells might be involved in the immune response in AC and MC.

The changes in cell types between AC and MC tissues and normal tissue in our findings involved mostly immune reactivity-related cells, highlighting the importance of immune regulation in AC and MC. Thus, we then analyzed the immune cell subclusters and their proportion in AC and MC tissues (Supplementary Figures S1A, B). Many immune cell types that represent an active immune environment were increased in AC and MC tissues compared with normal tissue. Such cells include CXCL13^+^ CD4^+^ T cells, which have antitumor effects in colon cancer (Ukita et al., 2022), CXCL13^+^ CD8^+^ T cells, and CXCL13^+^ CD8^+^ proliferating T cells. Gamma delta (γδ)-like PDCD^+^ T cells were also increased in AC and MC tissues compared with normal tissue; these cells have been reported to be enriched in deficient mismatch repair (dMMR) colon cancer (Pelka et al., 2021). CD4^+^ Treg and CD4^+^ proliferating regulatory T (Treg) cells were also increased in AC and MC tissues compared with normal tissue. These findings are consistent with results from previous studies that demonstrated that the infiltration of Treg cells into the colon was significantly higher in CRC tissue than in healthy colon tissue and that they had been associated with CRC progression (Allinen et al., 2004; Loddenkemper et al., 2006) (Supplementary Figure S1C). We then analyzed whether there were immune cells whose proportions differed between AC and MC tissues. We found that plasmacytoid dendritic cells (pDCs), PLZF^+^ proliferating T cells, CD16A^+^ NK cells, GZMK^+^ NK cells, and XCL^+^ NK cells were all increased in MC tissue compared with AC tissue (Supplementary Figure S1D). These findings indicate that NK cells might be more important in MC and could be a potential target for immune therapy for MC.

Taken together, these findings suggest the reshaping of the tumor microenvironment during the tumorigenesis of AC and MC, and we emphasize the importance of epithelial and stromal cell infiltration. CXCL14^+^ cancer-associated fibroblasts and CCL8 fibroblast-like cell proportions were higher in both AC and MC tissues than in normal colon tissue. NK cells might be a potential target for immune therapy for MC.

### Epithelial and stromal cell interactions in colorectal adenocarcinoma not otherwise specified, mucinous adenocarcinoma, and normal colon tissues

**FIGURE 6:**
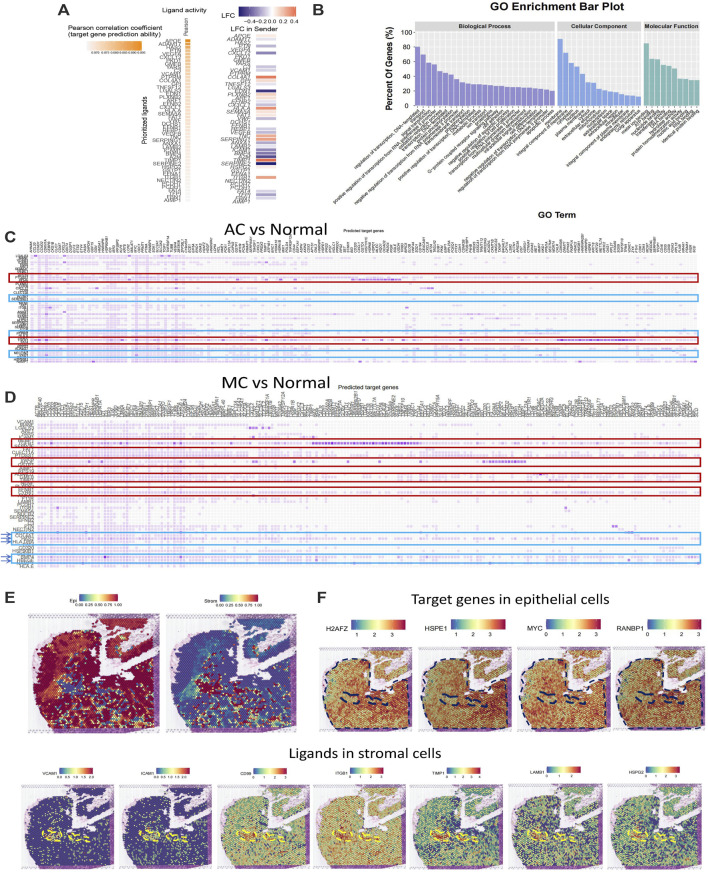
Stromal and epithelial cell interaction in human colorectal adenocarcinoma not otherwise specified, mucinous adenocarcinoma, and normal colon. **(A)** Top ligands in stromal cells regulating epithelial cells predicted by NicheNet from colorectal adenocarcinoma not otherwise specified and mucinous adenocarcinoma with normal colon as the reference. **(B)** GO enrichment of top ligands from colorectal adenocarcinoma not otherwise specified and mucinous adenocarcinoma with normal colon as the reference. **(C)** Top ligands in stromal cells predicted by NicheNet from colorectal adenocarcinoma not otherwise specified with normal colon as the reference and their target genes in epithelial cells. **(D)** Top ligands in stromal cells predicted by NicheNet from mucinous with normal colon as the reference and their target genes in epithelial cells. **(E)** Cell annotation on spatial transcriptome data on human colorectal adenocarcinoma not otherwise specified. **(F)** Spatial feature plot of target genes in epithelial cells and ligands in stromal cells predicted by NicheNet from colorectal adenocarcinoma not otherwise specified with normal colon as the reference.

Next, we used NicheNet (Browaeys et al., 2020) analysis to investigate potential ligands in stromal cells and target genes in epithelial cells. First, we analyzed the top ligands and target genes in both AC and MC tissues (Figure 6A). We then performed GO enrichment of the top ligands, and the biological process pathways in which the DEGs were enriched included the regulation of transcription, signal transduction, positive regulation of cell proliferation, and cell differentiation. The cellular component pathways in which the DEGs were enriched included integral components of the membrane, extracellular space, and extracellular region. The molecular function pathways included ATP, nucleotide, and RNA binding (Figure 6B). Thence, we analyzed the ligands in stromal cells and target genes in epithelial cells in AC and MC tissues and compared them with those of normal tissue (Figures 6C, D). We then analyzed the GO terms (Supplementary Figure S2A). The biological process pathways in which the DEGs between AC and normal tissues were enriched included cell adhesion, positive regulation of cell proliferation, and positive regulation of cell migration; these pathways were also observed in the comparison between MC and normal tissues. Positive regulation of the ERK1 and ERK2 cascade pathways was only enriched in the DEGs between MC and normal tissues, a finding which is consistent with previous research indicating that MC is associated with mutations in genes involved in the Ras-Raf-MEK-ERK pathway (Hugen et al., 2015b).

To further investigate the ligands on stromal and epithelial cells in AC and MC tissues, we calculated the top ligands of AC and MC tissues and compared them with those of normal colon tissue to determine whether there were overlapping and specific ligands of AC and MC tissues. Such ligands might be key molecules connecting stromal cells and tumor epithelial cells. Among the top 50 ligands of AC tissues and the top 48 ligands of MC tissues, 43 were overlapping ligands. Additionally, seven ligands were found only in AC tissue, and five only in MC tissue (Supplementary Table S4). Among the 43 overlapping ligands, apolipoprotein-E (APOE) and transforming growth factor (TGF)-β1 showed high prediction scores with target genes from epithelial cells, indicating that the two ligands might play important roles in stromal and epithelial cell interactions. Among the seven ligands that were specifically found in AC tissues, heparan sulfate proteoglycan 2 (HSPG2) (Datta et al., 2006), glycosylphosphatidylinositol (GPI) (Nicolas et al., 1994), and CD99 (Manara et al., 2018) were involved in regulating cell adhesion and invasion. Among the five ligands that were found only in MC tissues, bone morphogenetic protein 4 (BMP4) (Lombardo et al., 2011), hyaluronic acid synthase 2 (HAS2) (Kim et al., 2019), and heparin-binding epidermal growth factor-like growth factor (HBEGF) (Yun et al., 2018) were reported to be involved in cell proliferation or cell migration in colorectal cancer.

To further validate the ligand and target gene interactions between stromal and epithelial cells in colorectal cancer, we used spatial transcriptome data from an AC sample from Hu et al. (Peng et al., 2022). After running a routine process for spatial transcriptome data, including dimensionality reduction and clustering, we integrated the spatial transcriptome data with the single-cell data used in this study and annotated the cell type on the spatial plot. We observed that the stromal and epithelial cells were spatially connected to each other (Figure 6E). We then generated a spatial feature plot of ligands and target genes predicted from the comparison between AC and normal tissues. We found that the ligands VCAM1, ICAM1, CD99, ITGB1, TIMP1, LAMB1, and HSPG2 are highly expressed in the epithelial region and that their target genes of H2AFZ, HSPE1, MYC, and RANBP1 are highly expressed in the stromal region (Figure 6F).

In this section, we have sought to analyze the interactions between stromal cells and epithelial cells in AC and MC tissues and aimed to provide a potential therapeutic target for the more specific treatment of AC and MC. The positive regulation of the ERK1 and ERK2 cascade pathways was enriched only in DEGs between MC and normal tissues, indicating its potential for target development.

## Discussion

Regardless of improvements in therapeutic strategies for CRC patients, their overall prognosis is still not optimistic. With the development of biomarkers and drug targets, CRC treatment has become more individualized according to subtype classification with specific molecular and histological characteristics. With a higher proportion and more features of malignancy in young patients, the treatment and management of MC, as a unique subtype of CRC, have become a tough challenge in clinical practice. Although patients with MC account for a small proportion of CRC patients, they often suffer from poor prognoses, with the treatment of MC being a challenge for oncologists. Understanding molecular characteristics might impact clinical decisions for treatment strategies and promote the accurate treatment of CRC patients, especially with MC.

AC and MC are generally similar in tumor biological behavior. Cytochromes P450 (CYPs) are the major source of variability in drug response (Zanger and Schwab, 2013). We found that drug metabolism involving cytochrome P450 was inactive in AC and MC tissues. The dysfunction of CYP might be one of the reasons for poor drug response or even resistance in CRC.

Previous therapies have mainly depended on *KRAS* and *BRAF* mutations or MSI status, which are not pertinent to the MC subtype. This study aimed to identify more molecules and pathways that could be potential therapeutic targets for AC and MC.

It has been well-documented that hypoxia confers poor clinical outcomes *via* tumor propagation, malignant progression, and resistance to therapy (Hockel and Vaupel, 2001). Our proteomic analysis showed that cellular respiration-related biological processes were downregulated in both AC and MC tissues and that hypoxia-inducible factor-1 alpha signaling pathway-related proteins were upregulated in AC tissue compared to MC tissue. This finding reveals that, although hypoxia is a universal feature of tumor growth, it contributes more to the malignancy of AC but not MC. Therapy targeting hypoxia in the tumor environment would offer promising effects for AC but might not provide beneficial outcomes in AC treatment.

We found that the DNA damage response, which includes the CUL4B, RPS3, RPA1, and RPA2 proteins, was active in MC tissues, which might result in resistance to various therapies for MC. As an RNA-binding protein (RBP), RPS3 has been reported as a promising drug target for hepatocellular carcinoma (Zhao et al., 2019). The RPA (human replication protein A) family, including RPA1, RPA2, and RPA3, plays an essential role in eukaryotic DNA replication, homologous recombination, and excision repair; it has been recognized as an oncogene and therapeutic target for cancer (Chen et al., 2017; Wang et al., 2018).

The prevailing perspective is that the mucinous subtype elicits a worse response to immunotherapy than the non-mucinous subtype (Kim et al., 2013; Yan et al., 2022). Our analysis suggests that the positive regulation of type I interferon production and leukocyte-mediated immunity was enriched in the proteins upregulated only in AC tissue but not in MC tissue, while the leukocyte transendothelial migration pathway was inhibited in MC tissue. We infer that leukocyte transendothelial migration inhibition might be the major modality for insufficient leukocyte-mediated immunity and type I interferon production. Translocation-associated membrane protein 1 (TRAM1) was one of the most significantly upregulated proteins that were upregulated only in MC tissue. TRAM1 participates in human cytomegalovirus US2- and US11-mediated dislocation of an endoplasmic reticulum membrane glycoprotein and the subsequent degradation of major histocompatibility complex (MHC) class I heavy chains, thereby decreasing immune detection by cytotoxic T cells (Oresic et al., 2009). Together, both a lack of immune infiltration and MHC I binding led to immunotherapy resistance by MC and immunotherapy combined with targeting TRAM1 might improve the antitumor effect of immunotherapy for MC.

In addition, the proportion of bestrophin 4 (BEST4) ^+^ cell (Burclaff et al., 2022) was lower in AC and MC tissues than in normal tissue. A previous study reported that BMP3 was secreted by BEST4^+^ cells in the small intestine, with BMP3 being largely studied for its antitumor roles (Loh et al., 2008; Wen et al., 2019). Tuft cells are chemosensory cells that regulate type 2 immune reactions (Schneider et al., 2019) in the intestinal epithelial cell and could respond to pathogenic metabolites (Schneider et al., 2019). Therefore, BEST4^+^ and Tuft cells might act as potential targets for AC and MC immune therapy.

Apart from targeting cancer cells, anticancer therapies should target the stromal compartment to be effective and result in improved patient outcomes (Valkenburg et al., 2018). Previous studies have reported the pro-tumoral effects of CXCL14, based on evidence for the upregulation of CXCL14 in the tumor stroma and CAFs (Allinen et al., 2004; Augsten et al., 2009). In prostate cancer, CXCL14-producing fibroblasts have enhanced pro-tumoral effects (Augsten et al., 2009). In breast cancer, CCL8 is produced in stromal fibroblasts at the tumor margins and in tissues in which cancer cells tend to metastasize, such as the lungs and the brain (Farmaki et al., 2016). Therefore, CXCL14 fibroblasts and CCL8 fibroblast-like cells may have pro-tumorigenic functions in AC and MC. Interest has recently focused on NK cells for therapeutic interventions as these cells have antitumor properties (Chiossone et al., 2018). We found that NK cells might be important in MC and could be a potential target for immune therapy for MC.

Based on the different morphological characteristics, biological behaviors, and treatment responses (Johncilla and Yantiss, 2020), we explored the differences in cell types and functions between AC and MC. We found that there were few significantly different cell types and functions between AC and MC but noticed the important role of epithelial and stromal cells in CRC. We inferred that this difference contributed to the different biological behaviors and clinical outcomes of the two CRC subtypes. Moreover, we focused on ligands on stromal cells that might regulate the malignant behavior of tumor cells. Emerging evidence suggests that targeting the stromal compartment can shape the tumor environment and responsiveness to immunotherapy (Turley et al., 2015).

Apolipoprotein-E (APOE) and transforming growth factor (TGF)-β1 were identified as important ligands that interact between stromal cells and tumor epithelial cells and could be potential therapeutic targets for both AC and MC. APOE plays an important role in lipoprotein metabolism and is expressed at high levels by monocyte-derived macrophages (Mahley, 1988). It is highly expressed in colorectal liver metastases in colorectal cancer (Zhao et al., 2018). TGF-β1 is involved in almost every aspect of multipotent mesenchymal stromal cells (de Araújo Farias et al., 2018) and is shown to be a promoter of tumor progression and metastasis (Meulmeester and Ten Dijke, 2011). Furthermore, BMP4, which we identified as a specific ligand in MC, is a member of the TGF-β superfamily and is related to colorectal cancer progression (Li et al., 2022).

Collectively, we clarified the variations in the tumor environments among AC, MC, and normal colon tissues and tried to identify more molecular and cellular therapeutic targets for AC and MC.

However, our study does have certain limitations. First, the data source of proteomic, scRNAseq, and spatial transcriptome data belongs to different projects, which limited the analysis. It is difficult to warrant the sample information as comparable, such as sample gender, age, ethnicity, and race. Second, there has been until now no spatial transcriptome data on MC samples available to compare the spatial information between AC and MC. Third, the potential biomarkers or drug/therapy targets mentioned in this study are still hypothetical and need a lot of work to verify. We next aim to collect samples for proteomic, scRNAseq, and spatial transcriptome detection to further investigate this issue.

## Conclusion

There were different characteristics of the tumor environment between colorectal adenocarcinoma not otherwise specified (AC) and mucinous adenocarcinoma (MC). The major difference between AC and MC was not the variety of cell types and functions but may be cell interactions. Stromal and epithelial cell interactions play important roles in both MC and AC, but different regulatory pathways were involved.

## Data Availability

The original contributions presented in the study are included in the article/Supplementary Material; further inquiries can be directed to the corresponding authors.

## Supplementary material

SUPPLEMENTARY FIGURE S1
**(A)** Immune cell subcluster and proportion in human colorectal adenocarcinoma not otherwise specified, mucinous adenocarcinoma, and normal colon. **(B)** Bar plots showing the significance of different immune cell proportions that differed in two types of colorectal adenocarcinomas and normal colon. **(C)** Bar plots showing the significance of different immune cell proportions that differ in two types of colorectal adenocarcinoma.

SUPPLEMENTARY FIGURE S2
**(A)** GO enrichment of top ligands from colorectal adenocarcinoma not otherwise specified with normal colon as the reference, and from mucinous adenocarcinoma with normal colon as the reference.

## Abbreviations

AC, adenocarcinoma not otherwise specified; BP, biological process; CAFs, cancer-associated fibroblasts; CC, cellular component; CRC, colorectal cancer; DEGs, differentially expressed genes; GO, gene ontology; MC, mucinous adenocarcinoma; MF, molecular function; MSI, microsatellite instability; pDCs, plasmacytoid dendritic cells.
