# Supplementary figures and images for: Molecular characterization of colorectal mucinous adenocarcinoma and adenocarcinoma, not otherwise specified, identified by multiomic data analysis
Source: Front Mol Biosci. 2023 Apr 5;10:1150362. doi: 10.3389/fmolb.2023.1150362 (PMC10114614; doi:10.3389/fmolb.2023.1150362)

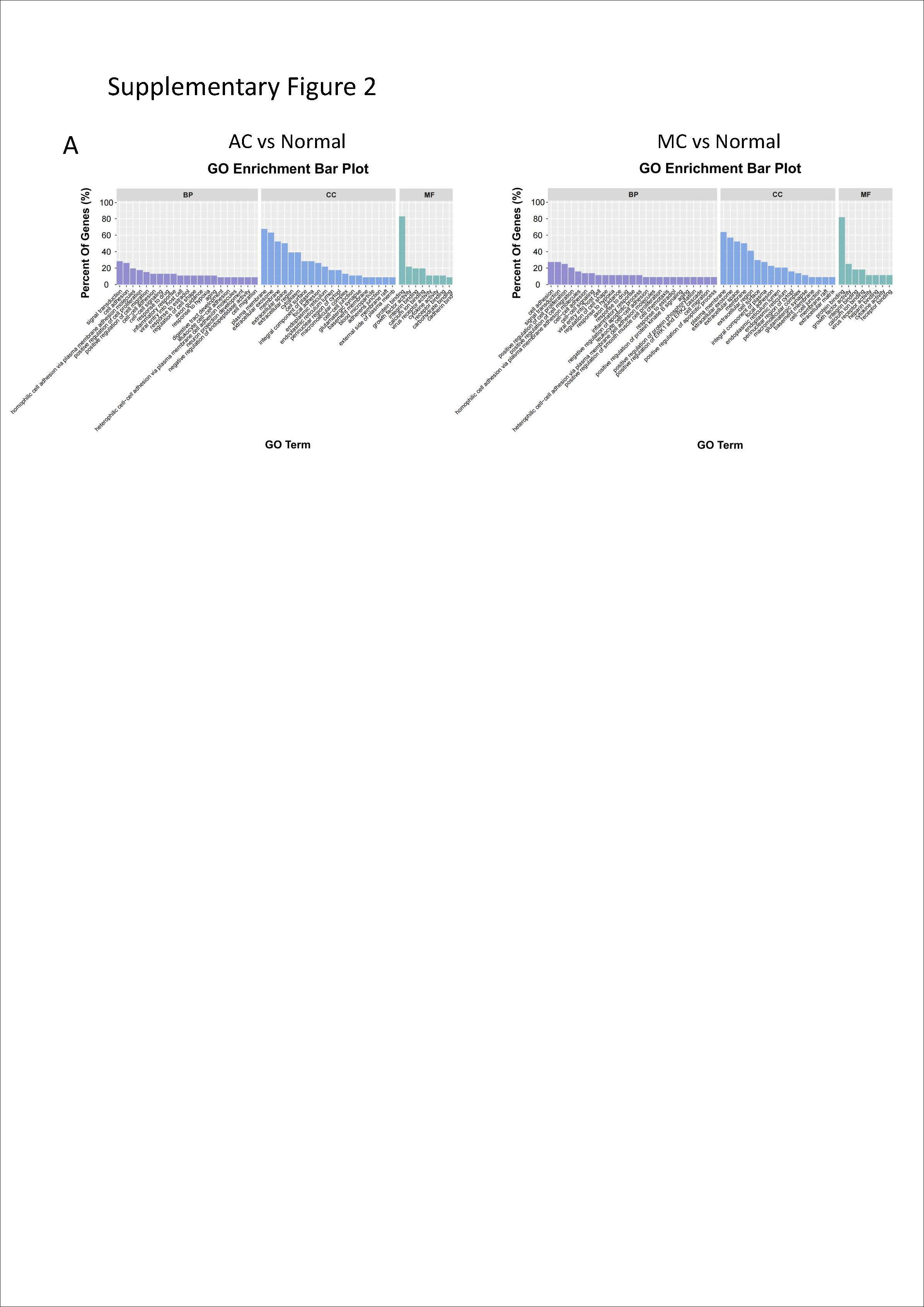

Supplement: Supplementary file 3 [file Image2.jpg]
